# How Brain Circuits Adapt to Changes in Sensory Experience

**DOI:** 10.1371/journal.pbio.1001802

**Published:** 2014-02-25

**Authors:** Janelle Weaver

**Affiliations:** Freelance Science Writer, Carbondale, Colorado, United States of America

The cerebral cortex, the outer portion of the brain, consists of a layered structure of neural tissue that contains the cell bodies of neurons and plays a key role in perception and cognition. Although cortical circuits throughout the brain share similarities, the patterns of connections among neurons in different brain regions can vary widely. Yet, relatively little is known about how region-specific wiring patterns relate to information processing in the brain.

**Figure:**
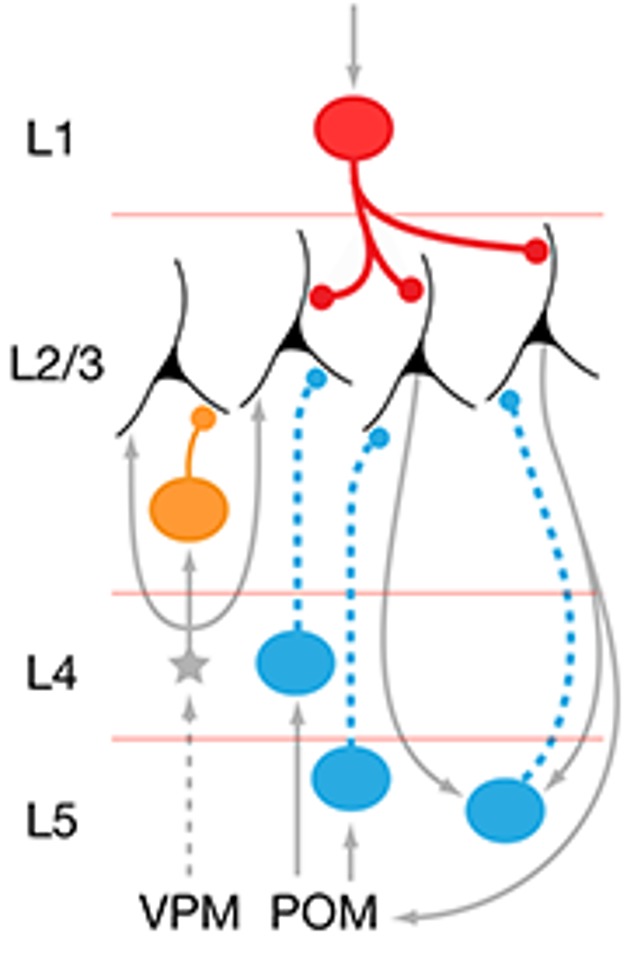
Specific neuronal connections can respond in independent and sometimes opposite ways to changes in sensory input.

In a study published in *PLOS Biology*, Dennis Kätzel and Gero Miesenböck of the University of Oxford investigated how wiring patterns in the brains of adult mice adapt to changes in sensory input. They found that distinct connections in neural networks responded in different ways to sensory changes, allowing neurons to continue to function optimally. The findings reveal a remarkable level of adaptability in cortical circuits of the adult brain.

To examine how brain circuits adapt to changes in sensory experience, the researchers used optogenetic mapping, a technique that combines genetics and optics to precisely control the activity of individual neurons. Kätzel and Miesenböck inserted channelrhodopsin, a light-sensitive receptor protein originally found in algae, into neurons, making them sensitive to activation by light of specific wavelengths. This allowed them to control the activity of these neurons and study their wiring patterns and function.

The researchers first examined how neural circuits would change in response to sensory deprivation. They trimmed the whiskers of adult mice to deprive the animals of tactile sensation for two to three weeks, and then prepared slices from barrel cortex, a brain region involved in processing tactile information from the whiskers. The researchers stimulated neurons in barrel cortex and then recorded light-evoked neural activity to map out neuronal connections. They found that sensory deprivation caused an overall reduction in inhibitory neural activity in cortical circuits, consistent with previous findings in brain regions that process visual information.

But this new study paints a more refined picture. Different neuronal connections responded in different ways to sensory deprivation. Neurons in layer 5 of barrel cortex stopped sending inhibitory signals to neurons in layer 2/3, making layer 2/3 neurons more excitable. This change may be an autoregulatory adaptation, because the lack of tactile signals from the whiskers had caused a sharp decrease in excitatory neural activity in barrel cortex. The release from inhibition could prevent a total shutdown of neural function, allowing neurons in layer 2/3 to continue to process even weak tactile signals in the wake of sensory deprivation.

On the other hand, neurons in layer 1 sent more inhibitory signals to neurons in layer 2/3 in response to sensory deprivation. Because layer 1 neurons carry information from higher brain regions involved in complex perceptual and cognitive processes, this adaptation could reflect top-down suppression of unusual activity in layer 2/3 neurons, which were no longer responding normally after sensory deprivation.

Moreover, these changes in cortical wiring patterns were entirely reversible. In a different experiment, the researchers trimmed the whiskers of adult mice for several weeks and then allowed the whiskers to regrow for four to five weeks before preparing the brain slices. The return to normal sensory stimulation restored the original level of inhibitory signals from neurons in layers 1 and 5 to neurons in layer 2/3. This finding suggests that changes in sensory stimulation do not destroy the underlying physical structure of neuronal connections, but rather modify the patterns of communication between neurons in cortical circuits.

Taken together, the findings suggest that specific neuronal connections can respond in independent and sometimes opposite ways to changes in sensory input. Surprisingly, these reversible changes occur in adulthood, well past the time window during which neuronal connections are thought to adapt to environmental fluctuations. The ability of wiring motifs to change in sophisticated ways could reflect a self-regulating mechanism that ensures optimal neural function in cortical circuits despite changes in sensory experience.

**Kätzel D, Miesenböck G (2014) Experience-Dependent Rewiring of Specific Inhibitory Connections in Adult Neocortex.** doi:10.1371/journal.pbio.1001798

